# Prokaryote-expressed M2e protein improves H9N2 influenza vaccine efficacy and protection against lethal influenza a virus in mice

**DOI:** 10.1186/1743-422X-10-104

**Published:** 2013-04-03

**Authors:** Eun-Ha Kim, Jun-Han Lee, Philippe Noriel Q Pascua, Min-Suk Song, Yun-Hee Baek, Hyeok-il Kwon, Su-Jin Park, Gyo-Jin Lim, Arun Decano, Mohammed YE Chowdhury, Su-Kyung Seo, Man Ki Song, Chul-Joong Kim, Young-Ki Choi

**Affiliations:** 1 Microbiology Department, College of Medicine and Medical Research Institute, Chungbuk National University, 12 Gaeshin-Dong Heungduk-Ku, Cheongju, 361-763, Republic of Korea; 2 College of Veterinary Medicine, Chungnam National University, 220 Gung-Dong, Yuseoung-Gu, Daejeon, 305-764, Republic of Korea; 3 Laboratory Science Division, International Vaccine Institute, Seoul, Republic of Korea

**Keywords:** Influenza A virus, M2e protein, Escherichia coli, Inactivated vaccine

## Abstract

**Background:**

Influenza vaccines are prepared annually based on global epidemiological surveillance data. However, since there is no method by which to predict the influenza strain that will cause the next pandemic, the demand to develop new vaccination strategies with broad cross-reactivity against influenza viruses are clearly important. The ectodomain of the influenza M2 protein (M2e) is an attractive target for developing a vaccine with broad cross-reactivity. For these reasons, we investigated the efficacy of an inactivated H9N2 virus vaccine (a-H9N2) mixed with M2e (1xM2e or 4xM2e) proteins expressed in *Escherichia coli*, which contains the consensus of sequence the extracellular domain of matrix 2 (M2e) of A/chicken/Vietnam/27262/09 (H5N1) avian influenza virus, and investigated its humoral immune response and cross-protection against influenza A viruses.

**Results:**

Mice were intramuscularly immunized with a-H9N2, 1xM2e alone, 4xM2e alone, a-H9N2/1xM2e, or a-H9N2/4xM2e. Three weeks post-vaccination, mice were challenged with lethal homologous (A/ chicken /Korea/ma163/04, H9N2) or heterosubtypic virus (A/Philippines/2/82, H3N2 and A/aquatic bird/Korea/maW81/05, H5N2). Our studies demonstrate that the survival of mice immunized with a-H9N2/1xM2e or with a-H9N2/4xM2e (100% survival) was significantly higher than that of mouse-adapted H9N2 virus-infected mice vaccinated with 1xM2e alone or with 4xM2e alone (0% survival). We also evaluated the protective efficacy of the M2e + vaccine against infection with mouse-adapted H5N2 influenza virus. Protection from death in the control group (0% survival) was similar to that of the 1×M2e alone and 4xM2e alone-vaccinated groups (0% survival). Only 40% of mice vaccinated with vaccine alone survived challenge with H5N2, while the a-H9N2/1×M2e and a-H9N2/4×M2e groups showed 80% and 100% survival following mouse-adapted H5N2 challenge, respectively. We also examined cross-protection against human H3N2 virus and found that the a-H9N2/1×M2e group displayed partial cross-protection against H3N2 (40% survival), whereas vaccine alone, 1×M2e alone, 4×M2e alone, or H9N2/1×M2e groups showed incomplete protection (0% survival) in response to challenge with a lethal dose of human H3N2 virus.

**Conclusions:**

Taken together, these results suggest that prokaryote-expressed M2e protein improved inactivated H9N2 virus vaccine efficacy and achieved cross-protection against lethal influenza A virus infection in mice.

## Background

Influenza A virus is an important human pathogen that causes occasional pandemics and has a huge impact on global health. Vaccination is the most economical and effective strategy by which to control the emergence and spread of influenza pandemics [1,2]. There are several influenza vaccines that have been licensed for use in humans, such as inactivated or live-attenuated whole virus vaccines, split vaccines, and subunit vaccines [1,3]. Inactivated seasonal vaccines include antigens from at least three different influenza strains. They are prepared annually in an effort to match vaccine composition with the global epidemiological surveillance data for a particular year [4,5]. Unfortunately, these vaccines are mainly designed to induce subtype-specific neutralizing antibodies and do not protect against infection with other influenza subtypes or with antigenic variants [4,6]. Additionally, because the influenza virus strain that will cause the next epidemic or pandemic cannot be predicted, new vaccination strategies that will result in broad cross-reactivity against influenza viruses need to be developed. The use of the ectodomain of the influenza virus matrix 2 protein (M2e) as an attractive target for developing broadly cross-reactive, universal influenza virus vaccines has been conceptualized and tested for several decades [7]. The M2e sequence is highly conserved across influenza virus subtypes (Table 1), and induced humoral anti-M2e immunity protects against lethal influenza virus challenge in animal models [8]. M2 vaccine candidates that have been explored included peptide-carrier conjugates [9], baculovirus-expressed M2e [10], fusion proteins [11,12], multiple antigenic peptides [13,14], and M2e DNA constructs that potentially express M2 [15,16]. In this regard, previous studies of M2e conjugate vaccines used various adjuvants such as Freund’s adjuvants [17], cholera toxin [18], heat labile endotoxins derivatives, flagellin [19], or bacterial protein conjugates [20]. These adjuvants or conjugates (viral particles or carrier molecules) [21], even combined with inactivated vaccine, were not completely protective against influenza virus infection as immunized animals still showed disease symptoms such as weight loss.

**Table 1:** Comparison of M2e sequence among vaccine and challenge strains

**M2e sequence homology**		
**M2e Protein**	A/ chicken /Vietnam/27262/2009 (H5N1)	MSLLTEVETPTRNEWECRCSDSSD
**Inactivated vaccine**	A/ chicken /Korea/04163/2004 (H9N2)	MSLLTEVETPTRN**G**WEC**K**CSDSSD
	A/chicken/Korea/ma163 (H9N2)	MSLLTEVETPTRN**G**WEC**K**CSDSSD
**Challenge virus**	A/aquatic bird/Korea/maW81/05 (H5N2)	MSLLTEVETPTRN**G**WEC**K**CSDSSD
	A/Philippines/82 (H3N2)	MSLLTEVETP**I**RNEW**G**CRC**N**DSSD

Amino acids in bold are the variant residues.

In this study, we investigated the efficacy of inactivated H9N2 virus vaccine (a-H9N2) mixed with 1×M2e or 4×M2e proteins expressed in *Escherichia coli* without adjuvant and were administered via the intramuscular route. Mice immunization and challenge experiments demonstrated that prokaryote-expressed M2e (1×M2e and 4×M2e) protein itself improved the efficacy of inactivated H9N2 virus vaccine and achieved cross-protection against lethal influenza A virus in mice.

## Results

### Vaccines containing M2e protein induced cross-reactive humoral immune response in mice

**Figure 1:**
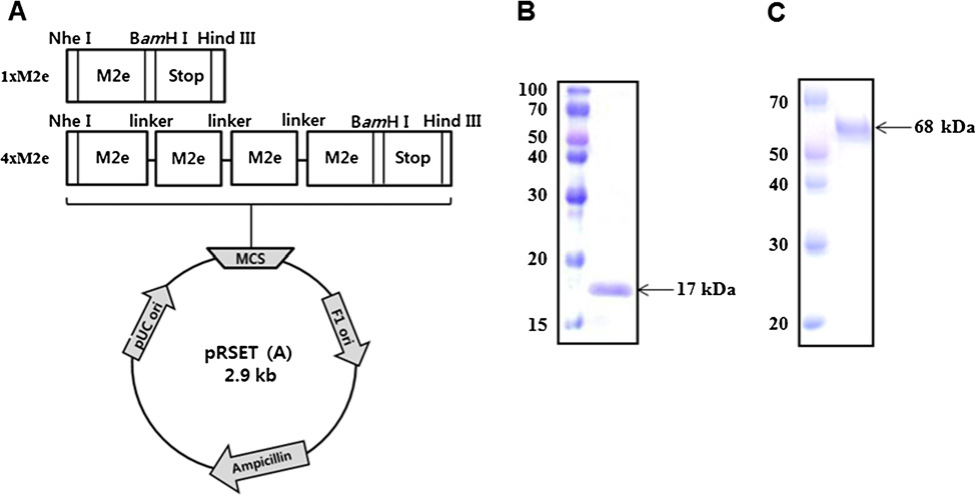
**Construction of plasmids and purification of 1×M2e or 4×M2e protein.** (**A**) The synthetic 1×M2e or 4×M2e genes from A/chicken/Vietnam/27262/09 (H5N1) were cloned into pRSETA vector. (**B**) Expression protein 1×M2e protein (17 kDa) and (**C**) 4×M2e protein (68 kDa) from *E. coli* cell, DE3.

**Figure 2:**
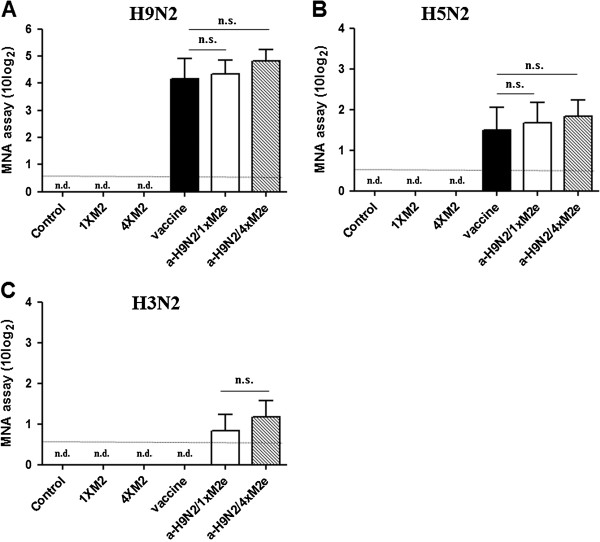
**M2e protein induces neutralization of influenza virus in mice.** 1×M2e, 4×M2e, a-H9N2, a-H9N2/1×M2e, or a-H9N2/1×M2e was used to immunize mice. Three weeks after boost vaccination, sera were collected. The samples were serially diluted two-fold. Serum neutralization activity was tested against 10^2^ TCID_50_/ml of respective viruses. (**A**) A/chicken/Korea/ma163/04 (ma163/H9N2), (**B**) A/aquatic bird/Korea/maW81/05 (maW81/ H5N2), or (**C**) A/Philippines/2/82 (Phil82/H3N2) virus for 30 min, followed by incubation with MDCK cells for 48 h. Data are representative of three independent experiments with three replicate wells per group. The lower limit of detection (0.5 10 log_2_TCID_50_) is indicated by a dotted line.

Two plasmid constructs bearing monomer or polymer of the viral M2e protein derived from A/chicken/Vietnam/27262/09 (H5N1) avian influenza virus (1×M2e and 4×M2e, respectively) were expressed in prokaryotic cells (BL21). Upon confirmation of protein e×pression and subsequent purification (Figure 1), groups of nineteen mice were intramuscularly (i.m.) immunized with 2 μg of inactivated whole-virus H9N2 vaccine (a-H9N2), only 1×M2e (15 μg), only 4×M2e (15 μg), inactivated H9N2 + 1×M2e (a-H9N2/1×M2e) and inactivated H9N2 + 4×M2e (a-H9N2 vaccine/4×M2e) with two doses at three week intervals. Polyclonal sera from immunized mice, taken three weeks after the first and second administration, were analyzed by hemagglutination inhibition (HI) test to identify IgG antibodies (Abs) directed against influenza A virus. Table 2 shows mice immunized with inactivated H9N2 vaccine (a-H9N2) developed a considerable antibody response against H9N2 virus, but not against H5N2 or H3N2 virus. In particular, HI titer specific only for homologous virus (ma163/04, H9N2) was noted in the mice immunized with a-H9N2 alone (245.11 HI titers) and a-H9N2/1×M2e (375.5 HI titers), and a-H9N2/4×M2e (929.55 HI titers) but not against heterosubtypic (maW81/05, H5N2 and Phil/82, H3N2) influenza viruses (Table 2); more appreciable homologous antibody titers were induced by a-H9N2/4×M2e (929.55 HI titers). To evaluate whether the noted antibodies could neutralize influenza virus, serum samples were tested by microneutralization assay (Figure 2). Among the M2e protein-mixed vaccines, receipt of the a-H9N2/4×M2e vaccine preparation induced neutralization titer relative to a-H9N2 alone against the H9N2 (4.16 versus 4.8 log_2_HAU) (*p* = 0.085) and H5N2 (1.5 versus 1.83 log_2_HAU) (*p* = 0.259) viruses. Interestingly, a-H9N2/4×M2e was able to neutralize the human Phil82/H3N2 virus although the polymer vaccine preparation remained most efficient (Figure 2C). None of the other vaccine groups could elicit detectable titers beyond the limit of detection.

**Table 2:** Hemagglutination inhibition antibody titer of sera collected 2 week after boost immunization

		**HI titers (GMT)**^**a**^	
	**H9N2**	**H5N2**	**H3N2**
**Control**	< 20	< 20	< 20
**1xM2e**	< 20	< 20	< 20
**4xM2e**	< 20	< 20	< 20
**Inactivated vaccine (H9N2)**	245.11	< 20	< 20
**1xM2e + vaccine**	375.5	< 20	< 20
**4xM2e + vaccine**	929.55	< 20	< 20

^a^ HI antibody titers were determined against A/chicken/Korea/ma163/04 (ma163/H9N2), A/aquatic bird/Korea/maW81/05 (maW81/H5N2), or A/Philippines/2/82 (Phil82/H3N2) viruses of the highest dilution of sera that inhibited hemagglutination by 4HA units of viruses. The results are the geometric mean titer of positive sera (≥20).

### 1×M2e or 4×M2e mixed with inactivated a-H9N2 vaccine induced protection against a mouse-adapted H9N2 avian influenza virus

**Figure 3:**
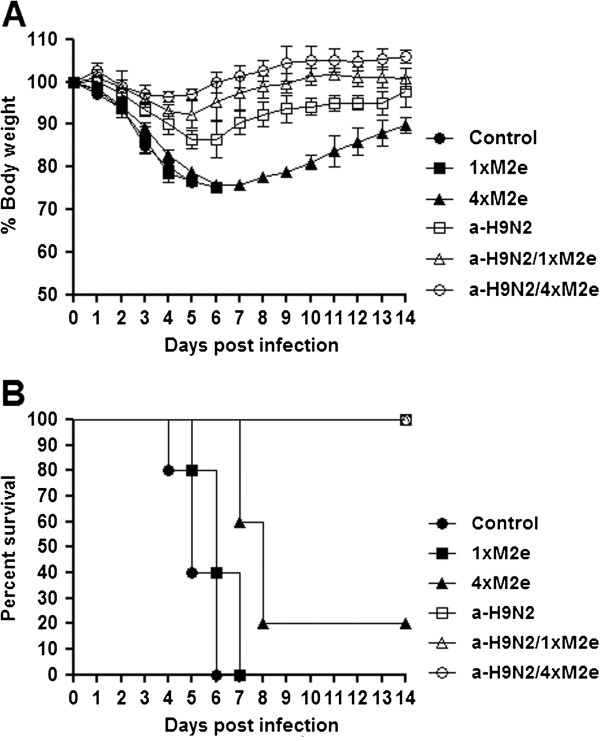
**Protection against homologous lethal challenge by addition of M2e protein to vaccine.** Groups of immunized mice and control mice were intranasally challenged with a lethal dose (2LD_50_) of A/chicken/Korea/ma163/04 (ma163/H9N2) influenza virus three weeks after boost vaccination. (**A**) Body weight changes and (**B**) survival were recorded for 14 days post-challenge. 1×M2e or 4×M2e mixed with inactivated H9N2 vaccine induced cross-protection against heterosubtypic avian H5N2 and human H3N2 influenza virus.

To determine whether prokaryote-expressed 1×M2e or 4×M2e proteins could improve the efficacy of the inactivated a-H9N2 vaccine derived from A/chicken /Korea/163/04 and confer protection against infection from a virulent mouse-adapted homologous variant virus, immunized mice were challenged with a lethal dose of A/chicken /Korea/ma163/04 (ma163/H9N2) virus two weeks after the last vaccination. Protective efficacy and morbidity (measured by survival rates and weight losses, respectively) were monitored every other day for 14 days post-infection (dpi); mice were euthanized and considered dead if the original body weight is reduced by >25%. Groups of mice administered with the purified protein alone (1×M2e vaccine groups), as well as mock-immunized group, showed weight losses (>25%) at 6 to 7 dpi resulting in a survival rate of 0% by 14 dpi. Some of 4×M2e alone-immunized mice had slightly extended mean survival relative to mice immunized with 1×M2e or mock-immunized animals (*p* = 0.39). In contrast, receipt of the a-H9N2 vaccine demonstrated moderate (14%) weight loss but conferred 100% survival in mice at 14 dpi. Interestingly, mice that were immunized with a-H9N2/1×M2e or with a-H9N2/4×M2e vaccine all survived (100%) until 14 dpi but were accompanied with very modest loss of body weight (3-7%) (Figure 3). These results demonstrate that the a-H9N2 vaccine itself could protect mice from lethal H9N2 virus infection whereas combination with the prokaryotic-expressed 1×M2e or 4×M2e protein moderated signs of morbidity and clinical disease.

**Figure 4:**
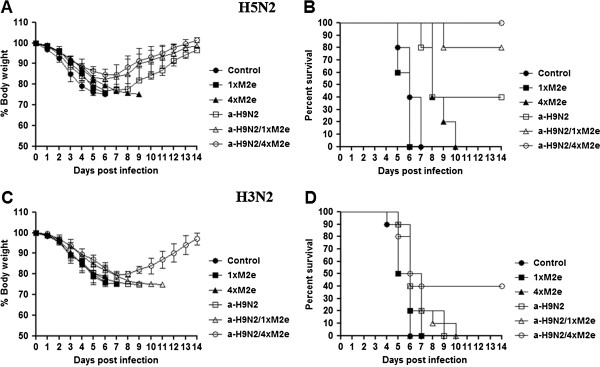
**Protective efficacy against a heterologous influenza viruses challenge.** Immunized mice were intranasally challenged with 2LD_50_ of an A/aquatic bird/Korea/maW81/05 (maW81/H5N2) influenza virus. Changes in body weight and survival were recorded daily post-challenge. (**A**) The average percent of initial weight is expressed as a percentage of the weight of the examined day relative to weight prior to challenge. (**B**) Survival was monitored for 14 days post-challenge. And mice were challenged with a lethal dose of A/Philippines/2/82 (Phil82/H3N2) influenza virus. (**C**) Body weight changes and (**D**) survival are shown.

M2e protein vaccination has been considered as a method to enhance cross protection against antigenic variants and even hetero-subtypes of influenza A viruses [20]. To better understand the degree of cross-protection of *E. coli*-expressed M2e protein in the context of the a-H9N2 vaccine, groups of vaccinated mice were lethally challenged by i.n. infection with a mouse-adapted avian H5N2 A/aquatic bird/Korea/maW81/05 (maW81/H5N2) [22] virus at two weeks after the last vaccination. Immunization with a-H9N2/1×M2e and a-H9N2/4×M2e induced high survival rates at 80% and 100%, with only 15-17% mean weight losses (Figure 4A and 4B). On the other hand, the group vaccinated with the inactivated a-H9N2 vaccine alone exhibited up to 20% reduction in body weight and at 7 dpi, only four out of ten mice survived the lethal infection (40% survival rate). The mock-vaccinated control, 1×M2e, and 4×M2e only vaccine groups displayed the highest weight losses (>25%) and all mice eventually succumbed to death within 6 to 10 dpi (Figure 4A and 4B). To further illustrate the breadth of cross-protection induced by the M2e protein, we also lethally challenged additional groups of immunized mice with a human H3N2 A/Philippines/82 virus (Phil82/H3N2) at two weeks post-vaccination. All mice in the control, 1×M2e only, 4×M2e only, and a-H9N2 vaccine groups became severely ill, lost weight (>25% from baseline) starting at 4 dpi, and all mice eventually died by 9 dpi (Figure 4C and 4D). Receipt of a-H9N2/1×M2e vaccine mix extended survival but could not completely protect immunized mice during the course of experiment. In contrast, the a-H9N2/4×M2e immunized group experienced 19% loss in body weight within 4 to 7 dpi, but started to recover thereafter (Figure 4C); 40% of the vaccinated mice survived the H3N2 virus challenge. Altogether, these results suggest that addition of the bacterially expressed M2e protein stimulated enhanced heterosubtypic protection even against a human Phil82/H3N2 virus despite some accompanying morbidity and mortality as reflected by body weight loss; improved results were markedly observed with the multimer 4×M2e protein mixture.

### 1×M2e and 4×M2e proteins induced viral clearance in mice challenged with lethal dose of influenza viruses

**Figure 5:**
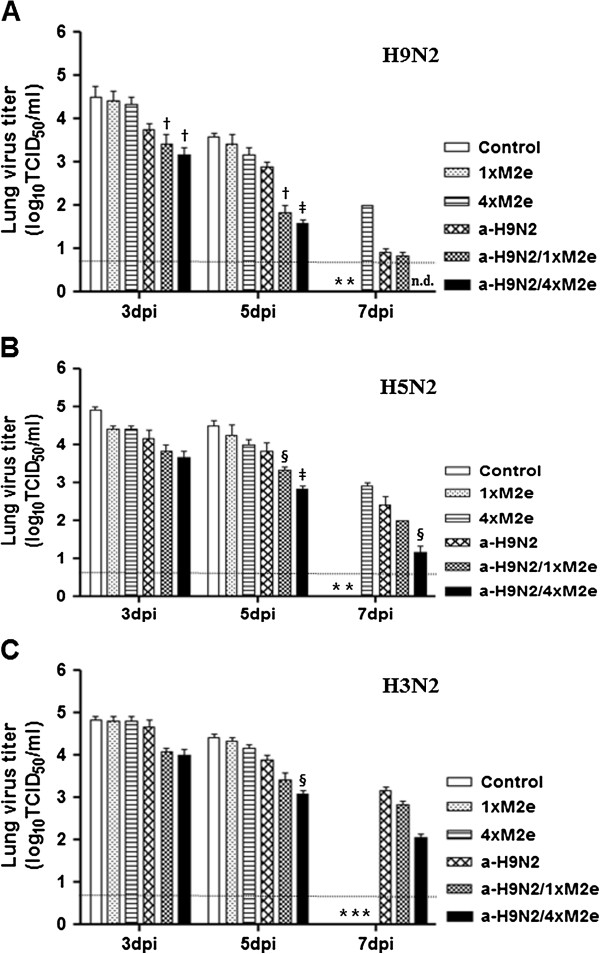
**Vaccine containing M2e protein showed a reduction in viral load during the course of influenza A virus infection.** BALB/c mice (9 heads/group) were infected with A/chicken/Korea/ma163/04 (ma163/H9N2), A/aquatic bird/Korea/maW81/05 (maW81/H5N2), or A/Philippines/2/82 (Phil82/H3N2) influenza virus at 2LD_50_ per mouse. Mice were sacrificed 3, 5, or 7 days post-challenge and samples were collected for lung virus titer. Lung (**A**) ma163/H9N2, (**B**) maW81/H5N2, and (**C**) Phil82/H3N2 influenza virus titers were detected at 3, 5, and 7 days post-challenge. The data are presented as GMT ± SD of 3 mice per group. ^†^ indicates *p* < 0.01 compared to the control group; ‡, *p* < 0.001 compared to the control group; §, *p* < 0.05 relative to the control group; mice died are indicated by *. The lower limit of detection (0.75 log_10_TCID_50_) is indicated by a dotted line.

We also assessed the ability of vaccines containing M2e proteins to inhibit viral growth in lungs of immunized and subsequently infected animals. Two weeks after the second administration, groups of immunized mice were challenged i.n. with 30 ul 10^5^ TCID_50_ of ma163/H9N2, maW81/H5N2, or Phil82/H3N2 virus. At 3, 5, and 7 dpi, lungs were collected from infected mice (3 heads per day) and MDCK cells were inoculated with supernatants from tissue homogenates for virus detection by TCID_50_ titration. The control and 1×M2e vaccine groups succumbed to infection. Receipt of the a-H9N2 vaccine moderately reduced lung viral titers up to 5 dpi but suppressed viral growth at 7 dpi relative to 4×M2e (2.0 versus 1.0 log_10_ TCID_50_/ml) (Figure 5A). More notably, immunization with the a-H9N2/1×M2e and a-H9N2/4×M2e vaccines demonstrated the most efficient inhibition of lung viral titers starting at 3 dpi and 7 dpi, none of the collected mice lungs produced virus titers beyond the limit of detection in the a-H9N2/4×M2e group. To provide additional assessment on cross-protective efficacy, groups of mice vaccinated with similar regimens were also challenged with heterosubtypic maW81/H5N2 and Phil82/H3N2 viruses at two weeks after the last immunization. As expected, all control groups could not limit growth of the two challenge viruses producing 4.5 log_10_ TCID_50_/ml peak titers; almost similar trends were also observed in groups of mice that received the only M2e proteins (Figure 5B and 5C). Although both of the a-H9N2/1×M2e and a-H9N2/4×M2e groups appeared to inhibit mice lung titers, a-H9N2/4×M2e demonstrated the most significant reduction in viral titers up to 5 dpi particularly against the maW81/H5N2 challenge virus compared to control group (*p* < 0.001). Vaccination with a-H9N2 reduced growth of the maW81/H5N2 and Phil82/H3N2 viruses but titers did not reach significant values (*p* = 0.37 and *p* = 0.29, respectively) compared to the a-H9N2/4×M2e vaccine group at 7 dpi. Altogether these results indicate that administration of the M2e alone could not efficiently suppress viral replication in vaccinated mice compared to when it is coupled with the inactivated a-H9N2 vaccine.

## Discussion

During the last decade, H9N2 avian influenza viruses circulated worldwide in poultry populations causing mild respiratory disease and reductions in egg production [23-26]. However, H9N2 viruses do not appear to replicate efficiently or cause severe disease until in April 1999 when two World Health Organization (WHO) reference laboratories independently confirmed the isolation of avian H9N2 influenza A (A/HK/1073/99) viruses for the first time in humans [27]. Following that year, another strain of H9N2 virus has been isolated repeatedly from the human population in mainland China [27,28]. Other reports also indicated continuous interspecies transmission of H9N2 avian influenza virus from avian to mammalian hosts [27,29]. Therefore, WHO declared H9N2 influenza virus as a potential candidate for the next influenza pandemic [30]. Currently available influenza virus vaccines only induce humoral immunity by boosting anti-influenza antibodies whose targets are limited to the surface glycoproteins, HA and NA [31]. Accordingly, contemporary universal influenza vaccines were developed mainly based on conserved sequences in M2, HA1, HA2, and NP proteins of the influenza virus [32]. Because it is highly conserved in all types of influenza A viruses, M2e has been studied as a universal influenza vaccine target. A number of studies with M2e vaccines have already been conducted [17,20,32-34] and recently, phase I clinical studies have been carried out with chemically or genetically produced M2e fusion proteins [35]. Tompkins *et al.*[36] proposed that various M2e sequences of M2 expression constructs could be used as vaccines. Despite substantial sequence divergence, H5-derived vaccines might also protect against circulating H1N1 and H3N2 subtypes. Here, we investigated the potential of vaccines containing prokaryotic expressed monomer or polymer of M2e proteins (1×M2e and 4×M2e, respectively) without adjuvant, to contribute to cross-protective immunity against several influenza virus subtypes. 1×M2e and 4×M2e clones were generated by using consensus M2e gene from an H5N1 avian virus without its trans-membrane domain (Table 1). In contrast to adjuvanted M2e vaccine studies, our serologic assays revealed that receipt of the prokaryotic cell-expressed M2e protein alone did not exhibit neutralizing activity against homologous or heterologous viruses indicating that our M2e formulation might not be sufficient to prevent morbidity. Similar results were also observed in a report that utilized baculovirus-expressed M2 VLPs [20]. Surprisingly, apart from providing homologous protection, an inactivated H9N2 (a-H9N2) vaccine in combination with the 4×M2e protein elicited enhanced cross-protection against a mouse-adapted H5N2 avian virus A/aquatic bird/Korea/maW81/05 and appeared to extend against a human H3N2 (A/Philippines/2/82) virus. Although sterile immunity was not achieved in any of our vaccination strategies, our data demonstrated potentially interesting enhancement in cross-protection.

Neutralization of influenza viruses has been primarily attributed as a function of antibodies directed against the HA surface glycoprotein antigen. However, anti-NA antibodies could also produce apparent neutralization by steric inhibition of virus adsorption and by interfering with viral release [37,38]. Comparison of the deduced N2 amino acid sequences of the three viruses showed 91.9% and 83.8% homology between H9N2 and the H5N2 and H3N2 viruses, respectively. Therefore, we could not completely rule out the role of N2-derived antibodies in the cross-neutralization and protection observed in Figures 2 and 4. Apparently though, mixture of the a-H9N2 vaccine with monomer and polymer M2e exhibited improved serologic and survival values particularly those with the a-H9N2/4×M2e vaccine group. M2e-specific antibodies have been shown to induce humoral immunity and mediate protection against influenza infection *in vivo*[39,40]. Furthermore, M2e-specific antibodies could promote antibody-dependent cell-mediated cytotoxicity (ADCC) and/or complement-mediated cytotoxicity (CDC) [41,42]. Therefore, we speculate that the difference in cross-protectivity afforded by the 1×M2e and 4×M2e, albeit administration of similar antigen concentrations, was mediated by the multiple copies of the M2e proteins that induced more robust cross-reactive antibodies.

Development of influenza M2e vaccines based on prokaryotic expression system without adjuvant is significant since *E. coli*-expressed M2e can be easily produced, safe and practical for animal and public health use. One concern about M2-based vaccines is the possibility that escape mutants may arise. However, a study of forced escape mutants found limited diversity [43] indicating that structural constraints, perhaps due to the requirements of the M1 structure encoded by the same segment, may limit drift [36]. It is noteworthy that our vaccination strategy showed that H9N2/4×M2e could protect the immunized host against a range of the viruses containing mismatched amino acid sequence (ranging from 0 to 3 out of 24 amino acids) of the M2e protein from A/chicken/Vietnam/27262/09 (H5N1) strain (Table 2). A previous report has shown that the monoclonal anti-M2e 14C2 (IgG1) antibody inhibits plaque growth of some influenza strains *in vitro*[10]*.* In addition, another study showed that M2 VLPs (eukaryotic expression system) provides complete cross protection against influenza A virus [20]. However, producing the VLP-based M2 proteins is relatively tedious and expensive compared to prokaryote-expressed ones. In addition, most of the studies conducted so far used M2e proteins in combination with various adjuvants. Therefore such reports may not have appreciated the additive effect of the M2e proteins (alone) which we observed when combined with an inactivated whole-virus vaccine.

## Conclusion

Recently, there have been some concerns regarding the possible emergence of a new influenza pandemic by avian H5N1, H9N2, and H3N2 variants. Furthermore, the number of reported cases of human infections with a novel triple reassortant A (H3N2)v (isolated from North American swine) [44] has been increasing since July 2012 [45,46] indicating a potential public health risk. Therefore, the development of universal influenza vaccines against various subtypes is urgently needed. In this study, we have demonstrated the efficacy of *E. coli*-expressed M2e proteins in providing cross-protection against lethal influenza virus infection. We provide evidence that an inactivated a-H9N2 vaccine containing M2e proteins could be potential candidate for inducing cross-protection, as shown against avian A/ chicken /Korea/ma163/04(H9N2) and A/Aquatic bird/Korea/maW81/05(H5N2) and human A/Philippines/2/82(H3N2) influenza viruses. The cross-reactivity and protective efficacy of the M2e protein suggests that polymer M2e protein, which in our case 4×M2e, could potentially promote protection against other influenza viruses.

Overall, our results demonstrate that prokaryote-expressed 1×M2e and 4×M2e protein immunization with an inactivated vaccine are efficacious against influenza A virus in mice. Although sterile immunity was not achieved in any of our vaccination strategies, our data demonstrated potentially interesting enhancement in cross-protection. These findings may offer an approach to control epidemic and pandemic influenza viruses.

## Materials and methods

### Construction of plasmids expressing 1×M2e or 4×M2e protein

The M2e protein from A/chicken/Vietnam/27262/09 (H5N1) was amplified and inserted into pRSETA vector (Invitrogen, CA, USA). For the 1×M2e gene cloning, M2e was amplified using a forward primer containing the *Nhe* I enzyme recognition site (Primer 1) and a reverse primer (Primer 3) bearing the *Bam*H I and *Hind* III sites with stop codons (TAATGA) in between (Figure 1 and Table 3). To create the M2e polymer construct, a forward primer containing the *Bgl* II enzyme recognition site (Primer 2) was used and paired with Primer 3. Amplicons from Primer 1/3 and Primer 2/3 were digested with corresponding *Nhe* I/*Bam*H I (Fragment 1) and *Bgl* II/ *Hind* III restriction enzymes (Fragment 2), respectively. The fragments were then ligated together with a T4 DNA ligase (Invitrogen, CA, USA) and inserted into a T-easy vector (Promega, Wisconsin, USA). Cloned product is further digested with *Bam*H I and then fused with Fragment 2. The process was repeated until a construct bearing four copies of the M2e protein (4×M2e) was produced with linker DNA sequences (21 nucleotide bases) in between the polymer. Upon confirmation, the two proteins (1×M2e and 4×M2e) were expressed in *E. coli* BL21 (DE3) bacterial cells and then purified by Ni-NTA beads through the His–tag affinity purification. The purified proteins were further processed to remove potential bacterially-derived endotoxin as previously described [34]. Briefly, the purified 1×M2e and 4×M2e proteins containing endotoxin were filtered through Polymixin B column kit (GenScript, USA). The endotoxin level of each protein was measured by the toxinsensor™ chromogenic limulus amebocyte lysate (LAL) endotoxin assay kit according to the manufacturer’s instructions (GenScript, USA). Endotoxin levels of the proteins were less than 0.18. Concentrations of eluted proteins were determined by Bradford Protein Assay Kit (Bio-rad). Thirty micrograms of the purified proteins were electrophoresed on a 10-15% SDS-PAGE and were visualized by Coomassie brilliant blue staining (Figure 1B and 1C). Purified proteins were stored at -80°C until use.

**Table 3:** The list and sequence of primers used for PCR analysis

**Primer number**	**Sequence**	**Length (base)**
1	5^′^-CTAGCTAGCATGTCATTATTAACA-3^′^	24
2	5^′^-GAAGATCTATGTCATTATTAACA-3^′^	23
3	5^′^-AAGCTT**TAATGA**GGATCCACCTGAACCACCTGAACCACCTGAACCACCTTCAAGTTC-3	57

Nucleotide bases in bold are the stop codons. Sequences of the used restriction enzyme sites are underlined.

### Mice and viruses

Five-week-old female BALB/c (H-2^d^) mice were purchased from SAMTAKO (Pyungteack, Korea). The A/chicken/Korea/ma163/04 (ma163/H9N2), A/aquatic bird/Korea/maW81/05 (maW81/H5N2), and A/Philippines/2/82 (Phil82/H3N2) were grown for two days at 37°C in the allantoic cavities of 10-day-old fertile chicken eggs. Clarified allantoic fluids were aliquoted and then stored at -70°C.

### Cell line

Madin-Darby Canine Kidney (MDCK) cells obtained from ATCC were maintained in EMEM (LONZA, Inc., Allendale, NJ) supplemented with 5% fetal bovine serum (LONZA, Inc.), 1% penicillin/streptomycin (Gibco-Invitrogen, Inc., Carlsbad, CA), and 1% non-essential amino acids (Gibco-Invitrogen, Inc.).

### Vaccination and challenge study

Five-week-old female inbred BALB/c mice were used for all experiments. Groups of 19 mice were intramuscularly (i.m.) immunized with 2 μg of inactivated H9N2 vaccine (a-H9N2), only 1×M2e (15 μg), only 4×M2e (15 μg), inactivated H9N2 + 1×M2e (a-H9N2/1×M2e) and inactivated H9N2 + 4×M2e (a-H9N2 vaccine/4×M2e) with two doses at three week intervals. Two weeks after the final immunization, mice were lightly anaesthetized and challenged intranasally (i.n.) with 2LD_50_ of A/chicken/Korea/ma163/04 (ma163/H9N2), A/aquatic bird/Korea/maW81/05 (maW81H5N2), or A/Philippines/2/82 (Phil82/H3N2) in a volume of 30 μl. Following infection, three mice were sacrificed 3, 5, and 7 dpi for lung viral titrations whereas the remaining ten mice were monitored daily for morbidity assessed by measuring body weight loss and survival for up to 14 dpi. Individual body weights were recorded for each mouse on various days post-infection.

### Hemagglutination inhibition (HI) test

Total lung homogenate samples were treated with receptor-destroying enzyme (RDE, Denka Seiken, Japan) at 37°C overnight, followed by heat-inactivation at 56°C for 30 min. RDE-treated lung samples were serially diluted two-fold and incubated with 25 μl of ma163/H9N2, maW81/H5N2, or Phil82/H3N2 virus in U-bottom microtiter plates (Nunc, Corning, NY) for 30 min, followed by incubation with 50 μl of 0.5% turkey red blood cells (tRBCs) for 30 min.

### Neutralizing assay

Twenty-five microliters of Phosphate buffer saline (PBS) was dispensed in a 96-well microplate. Heat-inactivated serum samples (at 25 ul volume) were added in the first wells and serially diluted two-fold. An equal volume (25 ul) of live influenza virus at a concentration of 10^2^ TCID_50_/ml was added to all samples. The mixture of sera and virus was incubated at 37°C for 1 h, loaded onto near confluent MDCK cells in a 96-well tissue culture plate, and incubated for two days at 37°C in 5% CO_2_. The plates were incubated for 2 days and the cytopathic effect was visually assessed using an inverted microscope. 50 μl of either cell supernant in U-bottom microtiter plate (Nunc, NY, USA), followed by incubation with 50 μl of 0.5% tRBCs for 30 min.

### Virus titers in lung tissues

To determine titers of infectious virus in lungs of infected mice, lung samples from three mice per group were collected 3, 5, or 7 dpi. Lung tissues from euthanized mice were aseptically extracted and homogenized in minimal essential medium (MEM). Antibiotics were added to achieve 10% (w/v) suspensions of lungs. Ten-fold serial dilutions of samples were added in quadruplicate to a monolayer of MDCK cells seeded in 96-well cell culture plates 18 h before infection, and allowed to absorb for 2 h at 37°C. Fresh medium was then added to the cells, which are incubated back at 37°C for 48 h. Virus cytopathic effect (CPE) was observed daily and the viral titer was determined by the hemagglutinin (HA) test as follows. Fifty μl of 0.5% tRBCs were added to 50 μl of cell culture supernatant and incubated at room temperature for 30 min. Wells showing HA activity were scored as positive. The virus titer was calculated by the Reed and Muench method [47] and expressed as log_10_TCID_50_/ml of lung tissue.

### Statistical analysis

The data were analyzed using GraphPad Prism version 5.00 for Windows (GraphPad Software, La Jolla, CA). *p* values of less than 0.05 (*p* < 0.05) were considered to be statistically significant.

### Ethics statement

The research protocol for the use of mice in this study were conducted in strict accordance and adherence to relevant policies regarding animal handling as mandated under the Guidelines for Animal Use and Care of the Korea Center for Disease Control (K-CDC) and was approved by the Medical Research Institute (approval number CBNU-IRB-2012-GM01). Animal care and use in an enhanced biosafety level 3 containment laboratory was approved by the Animal Experiment Committee of Bioleaders Corp. (permit number BLS-ABSL-12-010).

## Competing interests

The authors declare that they have no competing interests.

## Authors’ contributions

EHK, PNP, AD and YKC conceived the study and wrote the paper. EHK, YHB, JHL, HIK, SJP, GJL, MYEC and SKS performed the experiments. EHK, MSS, MKS, CJK and YKC analyzed the data. All authors have read and approved of the final manuscript.

## References

[B1] Shishkina LN Skarnovich MO Kabanov AS Sergeev AA Olkin SE Tarasov SA Belopolskaya MV Sergeeva SA Epstein OI Malkova EM Antiviral activity of Anaferon (pediatric formulation) in mice infected with pandemic influenza virus A(H1N1/09) Bull Exp Biol Med 2010 149 612 614 10.1007/s10517-010-1006-0 21165400

[B2] Pappaioanou M Highly pathogenic H5N1 avian influenza virus: cause of the next pandemic? Comp Immunol Microbiol Infect Dis 2009 32 287 300 10.1016/j.cimid.2008.01.003 19318178

[B3] Bramley AM Bresee J Finelli L Centers for Disease Control and Prevention (CDC) Pediatric influenza Pediatr Nurs 2009 35 335 345 20166462

[B4] Kawai N Ikematsu H Hirotsu N Maeda T Kawashima T Tanaka O Yamauchi S Kawamura K Matsuura S Nishimura M Clinical effectiveness of oseltamivir and zanamivir for treatment of influenza A virus subtype H1N1 with the H274Y mutation: a Japanese, multicenter study of the 2007–2008 and 2008–2009 influenza seasons Clin Infect Dis 2009 49 1828 1835 10.1086/648424 19911968

[B5] Nichol KL Treanor JJ Vaccines for seasonal and pandemic influenza J Infect Dis 2006 194 Suppl 2 S111 S118 17163383 10.1086/507544

[B6] Duan S Boltz DA Seiler P Li J Bragstad K Nielsen LP Webby RJ Webster RG Govorkova EA Oseltamivir-resistant pandemic H1N1/2009 influenza virus possesses lower transmissibility and fitness in ferrets PLoS Pathog 2010 6 e1001022 10.1371/journal.ppat.1001022 20686654 PMC2912389

[B7] Ebrahimi SM Tebianian M Influenza A viruses: why focusing on M2e-based universal vaccines Virus Genes 2011 42 1 8 10.1007/s11262-010-0547-7 21082230

[B8] Wu F Huang JH Yuan XY Huang WS Chen YH Characterization of immunity induced by M2e of influenza virus Vaccine 2007 25 8868 8873 10.1016/j.vaccine.2007.09.056 18061317

[B9] Misplon JA Lo CY Gabbard JD Tompkins SM Epstein SL Genetic control of immune responses to influenza A matrix 2 protein (M2) Vaccine 2010 28 5817 5827 10.1016/j.vaccine.2010.06.069 20600476

[B10] Zebedee SL Lamb RA Influenza A virus M2 protein: monoclonal antibody restriction of virus growth and detection of M2 in virions J Virol 1988 62 2762 2772 2455818 10.1128/jvi.62.8.2762-2772.1988 PMC253710

[B11] Fan J Liang X Horton MS Perry HC Citron MP Heidecker GJ Fu TM Joyce J Przysiecki CT Keller PM Preclinical study of influenza virus A M2 peptide conjugate vaccines in mice, ferrets, and rhesus monkeys Vaccine 2004 22 2993 3003 10.1016/j.vaccine.2004.02.021 15297047

[B12] Slepushkin VA Katz JM Black RA Gamble WC Rota PA Cox NJ Protection of mice against influenza A virus challenge by vaccination with baculovirus-expressed M2 protein Vaccine 1995 13 1399 1402 10.1016/0264-410X(95)92777-Y 8578816

[B13] Frace AM Klimov AI Rowe T Black RA Katz JM Modified M2 proteins produce heterotypic immunity against influenza A virus Vaccine 1999 17 2237 2244 10.1016/S0264-410X(99)00005-5 10403591

[B14] Livingston BD Higgins D Van NG Evolving strategies for the prevention of influenza infection: potential for multistrain targeting BioDrugs 2006 20 335 340 10.2165/00063030-200620060-00003 17176120

[B15] Neirynck S Deroo T Saelens X Vanlandschoot P Jou WM Fiers W A universal influenza A vaccine based on the extracellular domain of the M2 protein Nat Med 1999 5 1157 1163 10.1038/13484 10502819

[B16] Mozdzanowska K Feng J Eid M Kragol G Cudic M Otvos L Jr Gerhard W Induction of influenza type A virus-specific resistance by immunization of mice with a synthetic multiple antigenic peptide vaccine that contains ectodomains of matrix protein 2 Vaccine 2003 21 2616 2626 10.1016/S0264-410X(03)00040-9 12744898

[B17] Zhao G Sun S Du L Xiao W Ru Z Kou Z Guo Y Yu H Jiang S Lone Y An H5N1 M2e-based multiple antigenic peptide vaccine confers heterosubtypic protection from lethal infection with pandemic 2009 H1N1 virus Virol J 2010 7 151 10.1186/1743-422X-7-151 20624292 PMC2912260

[B18] Yang XF Jiang Y Li WY Kuang Y Jiang ZH Wang FP Li MY Expression and immunity of fused protein H1N1 M2e and cholera toxin B Xi Bao Yu Fen Zi Mian Yi Xue Za Zhi 2008 24 263 266 18328189

[B19] Huleatt JW Nakaar V Desai P Huang Y Hewitt D Jacobs A Tang J McDonald W Song L Evans RK Potent immunogenicity and efficacy of a universal influenza vaccine candidate comprising a recombinant fusion protein linking influenza M2e to the TLR5 ligand flagellin Vaccine 2008 26 201 214 10.1016/j.vaccine.2007.10.062 18063235

[B20] Song JM Wang BZ Park KM Van RN Quan FS Kim MC Jin HT Pekosz A Compans RW Kang SM Influenza virus-like particles containing M2 induce broadly cross protective immunity PLoS One 2011 6 e14538 10.1371/journal.pone.0014538 21267073 PMC3022578

[B21] Quan FS Kim Y Lee S Yi H Kang SM Bozja J Moore ML Compans RW Viruslike particle vaccine induces protection against respiratory syncytial virus infection in mice J Infect Dis 2011 204 987 995 10.1093/infdis/jir474 21881112 PMC3164432

[B22] Song MS Pascua PN Lee JH Baek YH Lee OJ Kim CJ Kim H Webby RJ Webster RG Choi YK The polymerase acidic protein gene of influenza a virus contributes to pathogenicity in a mouse model J Virol 2009 83 12325 12335 10.1128/JVI.01373-09 19793828 PMC2786751

[B23] Swayne DE Beck JR Heat inactivation of avian influenza and Newcastle disease viruses in egg products Avian Pathol 2004 33 512 518 10.1080/03079450400003692 15545031

[B24] King DJ Evaluation of different methods of inactivation of Newcastle disease virus and avian influenza virus in egg fluids and serum Avian Dis 1991 35 505 514 10.2307/1591214 1835374

[B25] Thomas C Swayne DE Thermal inactivation of H5N1 high pathogenicity avian influenza virus in naturally infected chicken meat J Food Prot 2007 70 674 680 17388058 10.4315/0362-028x-70.3.674

[B26] Thomas C King DJ Swayne DE Thermal inactivation of avian influenza and Newcastle disease viruses in chicken meat J Food Prot 2008 71 1214 1222 18592748 10.4315/0362-028x-71.6.1214

[B27] Peiris M Yuen KY Leung CW Chan KH Ip PL Lai RW Orr WK Shortridge KF Human infection with influenza H9N2 Lancet 1999 354 916 917 10.1016/S0140-6736(99)03311-5 10489954

[B28] Guo YJ Krauss S Senne DA Mo IP Lo KS Xiong XP Norwood M Shortridge KF Webster RG Guan Y Characterization of the pathogenicity of members of the newly established H9N2 influenza virus lineages in Asia Virology 2000 267 279 288 10.1006/viro.1999.0115 10662623

[B29] Maines TR Szretter KJ Perrone L Belser JA Bright RA Zeng H Tumpey TM Katz JM Pathogenesis of emerging avian influenza viruses in mammals and the host innate immune response Immunol Rev 2008 225 68 84 10.1111/j.1600-065X.2008.00690.x 18837776

[B30] Choi YK Ozaki H Webby RJ Webster RG Peiris JS Poon L Butt C Leung YH Guan Y Continuing evolution of H9N2 influenza viruses in Southeastern China J Virol 2004 78 8609 8614 10.1128/JVI.78.16.8609-8614.2004 15280470 PMC479067

[B31] Moscona A Neuraminidase inhibitors for influenza N Engl J Med 2005 353 1363 1373 10.1056/NEJMra050740 16192481

[B32] Du L Zhou Y Jiang S Research and development of universal influenza vaccines Microbes Infect 2010 12 280 286 10.1016/j.micinf.2010.01.001 20079871

[B33] Moscona A Neuraminidase inhibitors for influenza N Engl J Med 2005 353 1363 1373 10.1056/NEJMra050740 16192481

[B34] Shim BS Choi YK Yun CH Lee EG Jeon YS Park SM Cheon IS Joo DH Cho CH Song MS Sublingual immunization with M2-based vaccine induces broad protective immunity against influenza PLoS One 2011 6 e27953 10.1371/journal.pone.0027953 22140491 PMC3227615

[B35] Schotsaert M De FM Fiers W Saelens X Universal M2 ectodomain-based influenza A vaccines: preclinical and clinical developments Expert Rev Vaccines 2009 8 499 508 10.1586/erv.09.6 19348565 PMC2706389

[B36] Tompkins SM Zhao ZS Lo CY Misplon JA Liu T Ye Z Hogan RJ Wu Z Benton KA Tumpey TM Matrix protein 2 vaccination and protection against influenza viruses, including subtype H5N1 Emerg Infect Dis 2007 13 426 435 10.3201/eid1303.061125 17552096 PMC2725899

[B37] Webster RG Laver WG Kilbourne ED Reactions of antibodies with surface antigens of influenza virus J Gen Virol 1968 3 315 326 10.1099/0022-1317-3-3-315 5711432

[B38] Webster RG Laver WG Preparation and properties of antibody directed specifically against the neuraminidase of influenza virus J Immunol 1967 99 49 55 6029282

[B39] De FM Fiers W Martens W Birkett A Ramne A Lowenadler B Lycke N Jou WM Saelens X Centers for Disease Control and Prevention (CDC) Improved design and intranasal delivery of an M2e-based human influenza A vaccine Vaccine 2006 24 6597 6601 10.1016/j.vaccine.2006.05.082 16814430

[B40] Mozdzanowska K Zharikova D Cudic M Otvos L Gerhard W Roles of adjuvant and route of vaccination in antibody response and protection engendered by a synthetic matrix protein 2-based influenza A virus vaccine in the mouse Virol J 2007 4 118 10.1186/1743-422X-4-118 17974006 PMC2186315

[B41] Jegerlehner A Schmitz N Storni T Bachmann MF Influenza A vaccine based on the extracellular domain of M2: weak protection mediated via antibody-dependent NK cell activity J Immunol 2004 172 5598 5605 15100303 10.4049/jimmunol.172.9.5598

[B42] Subbarao K Joseph T Scientific barriers to developing vaccines against avian influenza viruses Nat Rev Immunol 2007 7 267 278 10.1038/nri2054 17363960 PMC7097526

[B43] Zharikova D Mozdzanowska K Feng J Zhang M Gerhard W Influenza type A virus escape mutants emerge in vivo in the presence of antibodies to the ectodomain of matrix protein 2 J Virol 2005 79 6644 6654 10.1128/JVI.79.11.6644-6654.2005 15890902 PMC1112148

[B44] Nelson MI Vincent AL Kitikoon P Holmes EC Gramer MR Evolution of Novel Reassortant A/H3N2 Influenza Viruses in North American Swine and Humans, 2009–2011 J Virol 2012 86 8872 8878 10.1128/JVI.00259-12 22696653 PMC3421719

[B45] Lindstrom S Garten R Balish A Shu B Emery S Berman L Barnes N Sleeman K Gubareva L Villanueva J Human infections with novel reassortant influenza A(H3N2)v viruses, United States, 2011 Emerg Infect Dis 2012 18 834 837 10.3201/eid1805.111922 22516540 PMC3358066

[B46] Richards S House M Pontones P Metcalf D Marsh B Swenson S Korslund J Blanton L Epperson S Bigger M Outbreak of Influenza A (H3N2) Virus Among Persons and Swine at a County Fair - Indiana, July 2012 MMWR Morb Mortal Wkly Rep 2012 61 561 22832938

[B47] Reed LJ Muench H A simple method of estimating fifty per cent endpoints Am J Epidemiol 1938 27 493 497

